# Pea protein isolate modified by Persian gum and ultrasound treatment as a thickening agent for dysphagia-oriented instant rice porridge

**DOI:** 10.1016/j.ultsonch.2026.108083

**Published:** 2026-09-26

**Authors:** Yasamin Damroudi, Mehdi Farhoodi, Leila Mirmoghtadaie, Kooshan Nayebzadeh, Fereshte Bahmanyar

**Affiliations:** a Department of Food Science and Technology, National Nutrition and Food Technology Research Institute, Faculty of Nutrition Sciences and Food Technology, Shahid Beheshti University of Medical Sciences, Tehran, Iran; b Pistachio Safety Research Center, Rafsanjan University of Medical Sciences, Rafsanjan, Iran

**Keywords:** Dysphagia, Pea protein isolate, Ultrasound, Persian gum, Texture-modified foods

## Abstract

In this study, pea protein isolate (PPI) was modified using two strategies: complexation with Persian gum and ultrasound treatment. Pea protein isolate–Persian gum complexes (PPI–PG) were prepared at mass ratios of 4:1 and 2:1, and ultrasound-treated pea protein isolates (UPPIs) were obtained by subjecting PPI dispersions to ultrasound treatment at two power levels (100 and 300 W) for 10 and 20 min. The structural and functional properties of the modified PPIs (MPPIs) were characterized, followed by evaluation of their potential as functional thickening agents in instant rice porridge (IRP) for dysphagia management. Both modifications increased the solubility and surface charge of PPI and altered its water-holding capacity (WHC). PPI–PG complexes exhibited larger particle sizes, whereas UPPIs showed reduced particle sizes. However, prolonged ultrasound treatment at 300 W for 20 min adversely affected these properties. FTIR spectroscopy also revealed changes in the secondary structure of the MPPIs. IRPs containing MPPIs exhibited higher WHC and enhanced rheological and textural properties than porridges prepared with native PPI. Compared with PPI–PG complexes, UPPIs led to greater increases in WHC and textural properties of the reconstituted IRPs. SEM further revealed a more structured 3D network in UPPI-thickened porridges. Based on International Dysphagia Diet Standardisation Initiative (IDDSI) tests, porridges containing UPPI treated at 100 W for 10 and 20 min met the requirements for Level 4 and exhibited the best sensory characteristics. These findings highlight the potential of MPPIs, particularly UPPIs, as effective thickening agents in texture-modified IRPs for individuals with dysphagia.

## Graphical abstract

**Figure:**
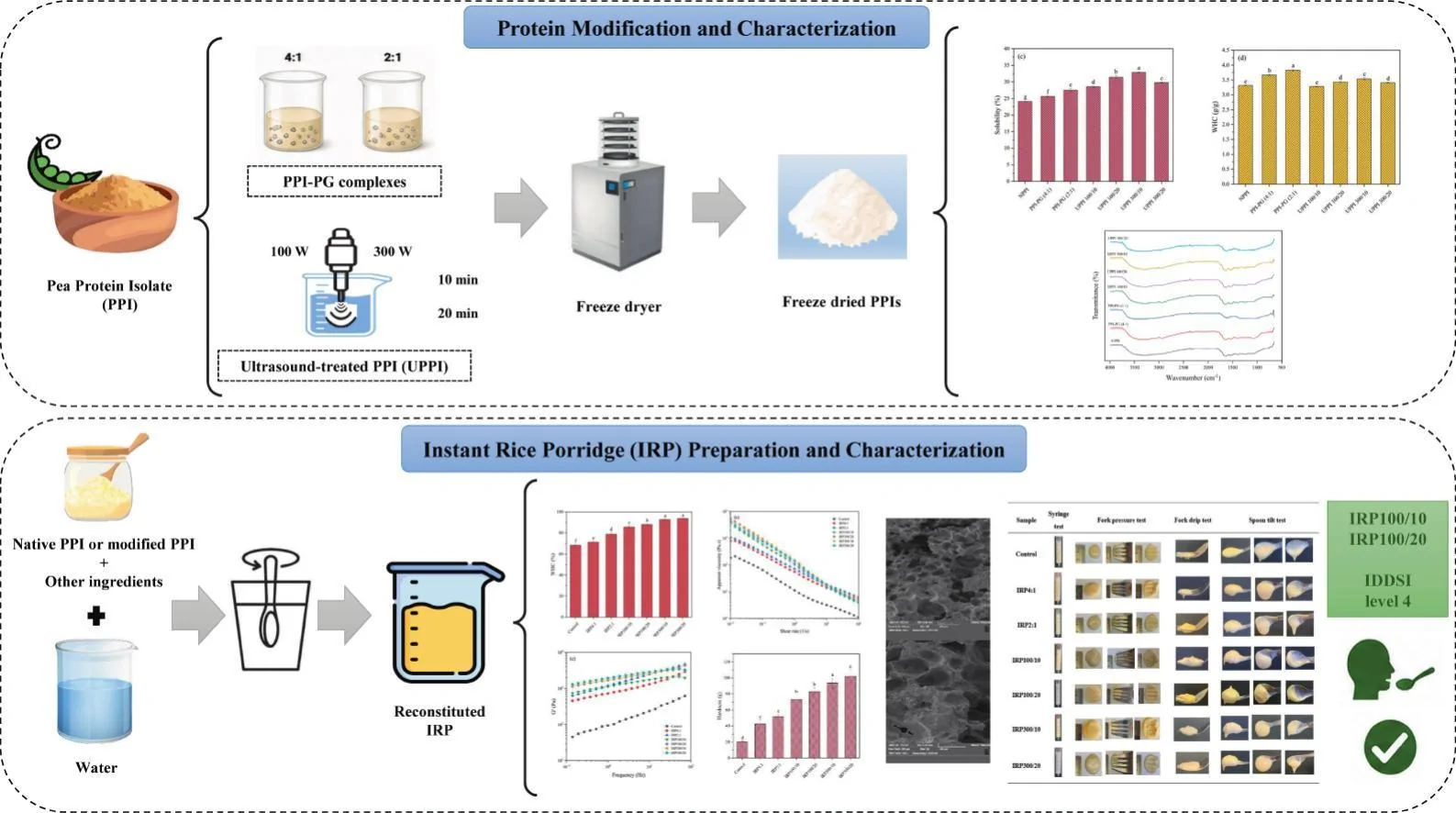

## 1 Introduction

Food consumption is one of the most pleasant aspects of normal daily life. Dysphagia is a medical condition that impairs an individual's ability to chew and swallow food, affecting both physical and psychological health. It is particularly prevalent among older adults and may also occur as a consequence of neurological and muscular disorders. Recent reports have also indicated an increased susceptibility to dysphagia among individuals affected by COVID-19. Dysphagia can result in choking, aspiration pneumonia, and reduced food intake, thereby compromising nutritional status and increasing the risk of malnutrition, weight loss, and sarcopenia [1], [2]. Approximately 8 % of the global population is affected by dysphagia, and its prevalence is expected to increase with population aging, making it an important healthcare concern [1], [3]. Among the various approaches to dysphagia management, texture-modified foods (TMFs) are widely used because they facilitate the formation of a cohesive and easily swallowable bolus, thereby reducing chewing effort and minimizing the risk of aspiration during swallowing [4], [5]. Therefore, foods intended for individuals with dysphagia should provide adequate nutritional quality and appropriate textural properties to ensure safe swallowing [6]. In this context, the International Dysphagia Diet Standardisation Initiative (IDDSI) provides a standardized framework for the classification and safety assessment of texture-modified foods and thickened liquids for dysphagia management [7].

Instant rice porridge represents a suitable food matrix for dysphagia-oriented formulations due to its soft texture, ease of preparation, extended shelf life, and convenience during transportation and distribution. However, as a rice-based product, it is primarily carbohydrate-rich and generally contains limited amounts of protein [8]. Accordingly, protein fortification is required to improve its nutritional quality. This is particularly important for older adults, as adequate protein intake may help maintain muscle mass and reduce the risk of sarcopenia [9]. In this regard, pea protein isolate (PPI) is a promising candidate, as it can enhance the protein content of the formulation while also contributing to texture development as a non-polysaccharide thickening ingredient.

PPI has received increasing attention due to its favorable amino acid profile, high lysine content, absence of major allergenicity and transgenic safety concerns, as well as its desirable techno-functional properties. However, it also exhibits several functional drawbacks, including poor solubility, reduced gelation and emulsification capacities [10], [11]. Protein–polysaccharide complexation and ultrasound treatment can effectively enhance the functional properties of proteins. Protein–polysaccharide interactions have been shown to enhance protein functionality by improving solubility and water-holding capacity (WHC) [12]. Furthermore, hydrocolloids play an important role in dysphagia-oriented foods by modifying rheological and mechanical properties to facilitate safe swallowing [13]. Persian gum (PG) is a natural anionic polysaccharide obtained from the exudate of the mountain almond tree (*Amygdalus scoparia Spach*). It is low-cost, has prebiotic potential, and is widely produced and distributed in Iran. PG has a high water absorption capacity and can increase the viscosity of the continuous phase [14], [15]. PG dispersions have also been reported to exhibit shear-thinning flow behavior at different concentrations and temperatures [16], which could be beneficial for swallowing in individuals with dysphagia [17]. Additionally, its hydroxyl and carboxyl groups contribute to its surface charge [14]. Together, these properties make PG a potential candidate for forming protein–polysaccharide complexes with PPI. In addition, ultrasound treatment (US), a safe and environmentally friendly technology, can be used as a physical method to modify protein properties. Cavitation generated during sonication disrupts protein aggregates, leading to their size reduction and resulting in structural changes that improve functional properties [18].

No study has yet investigated the use of PPI–PG complexes and ultrasound-treated PPI (UPPI) as thickening agents in dysphagia-oriented instant rice porridge. Therefore, in this study, the structural and functional properties of the modified pea protein isolates were first analyzed, followed by their addition into instant rice porridges. The resulting porridges were then evaluated for their rheological, textural, and sensory properties, and their suitability for dysphagia management was determined according to the IDDSI framework.

## 2 Materials and methods

### 2.1 Materials

PPI (85 % protein, 9.2 % fat, 0.1 % carbohydrates, 3.3 % fiber, and 1.5 % salt, based on dry weight) was obtained from the Fibrelle Company (Istanbul, Turkey). A single batch of commercial PPI from one supplier was used throughout the study to ensure experimental consistency and minimize potential batch-to-batch variability. PG was supplied from a local herbal market (Tehran, Iran). The white granules were first manually separated from all other-colored granules, after which the white granules were milled using a Moulinex blender (Paris, France). Milling was continued until the powder passed through a No. 70 mesh laboratory sieve with an aperture size of 212 µm (Damavand Co., Tehran, Iran), resulting in a uniform powder for subsequent experiments. Whey protein concentrate (WPC80) (80 % proteins, 5 % ash, 5 % fat, and 7 % lactose) was sourced from DMV Co. (Veghel, Netherlands). Skim milk powder (SMP) (34 % protein, 1.5 % fat, and 55 % carbohydrates) was obtained from Pegah Dairy Company (Hamedan, Iran). Rice flour (Golha, Iran) and additional ingredients incorporated into the instant rice porridge were purchased from local market suppliers.

### 2.2 Modification of pea protein isolate (PPI)

#### 2.2.1 Preparation of PPI–PG complexes

PPI-PG complexes were prepared by mixing PPI and PG in mass ratios of 4:1 and 2:1 (w/w) in distilled water at 10 wt % concentration. The pH of the solutions was adjusted to 7.0 using 1 N HCl or 1 N NaOH. The mixtures were then stirred on a magnetic stirrer at ambient temperature for 4 h to facilitate complete dissolution and promote interaction between PPI and PG. The samples were named PPI-PG (4:1) and PPI-PG (2:1), respectively.

#### 2.2.2 Preparation of ultrasound-treated PPI (UPPI)

Appropriate amounts of PPI powder were dispersed in distilled water to obtain 10% (w/w) protein dispersions. The samples were then stirred at 700 rpm using a magnetic stirrer for 1 h at ambient temperature (pH 7.0). Subsequently, 100 mL of the PPI dispersions were treated with a 20 kHz ultrasonic probe (UP-400-A, Iran) at two power levels (100 and 300 W) for 10 and 20 min using a pulse mode of 3 s on and 2 s off. The resulting samples were named UPPI 100/10, UPPI 100/20, UPPI 300/10, and UPPI 300/20. Untreated native PPI was coded as NPPI.

To prevent overheating during sonication, the temperature was maintained below 30 °C using an ice-water bath. Finally, the samples were lyophilized, milled, and stored at 4 °C until further analysis and use in instant rice porridge preparation.

### 2.3 Characteristics of the modified pea protein isolate (MPPI)

#### 2.3.1 Particle size distribution and zeta potential

The particle size and zeta potential (ζ) of the PPI dispersions were determined by dynamic light scattering (DLS) using a Zetasizer Nano ZS (Malvern Co., UK). Freeze-dried PPI powders were dispersed in distilled water at a concentration of 5 mg/mL prior to analysis.

#### 2.3.2 Solubility

Solubility was measured according to the method described by Bahmanyar et al. [18].

#### 2.3.3 Water-holding capacity (WHC)

10 ml distilled water with 1 g of samples were mixed in the pre-weighed centrifuge tube, followed by vortexing for 1 min. The suspensions was allowed to stand 30 min at rest. Subsequently, the mixtures were centrifuged for 30 min at 7080 × g. The supernatant was thrown away and let the residue was drained for 10 min before being weighed. WHC was calculated according to the Eq. (1) [18]:

*WHC (g/g)* = (*Amount of water absorbed by the PPI (g)/ Mass of the original PPI sample* (*g*)) (1)

#### 2.3.4 Fourier-transform infrared spectroscopy (FTIR)

FTIR spectra of lyophilized native and modified PPI samples were recorded using an Agilent Cary 630 FTIR spectrometer (Agilent Technologies, Santa Clara, CA, USA) equipped with a diffuse reflectance (DRIFTS) module. The spectra were collected over the wavenumber range of 4000–650 cm^−1^ using 60 sample scans and 20 background scans at a resolution of 8 cm^−1^. All measurements were performed at room temperature [18]. To evaluate protein secondary structure, the amide I region (1700–1600 cm^−1^) was deconvoluted using Gaussian curve fitting in OriginPro 2024 (OriginLab, USA). The relative percentages of α-helix, β-sheet, β-turn, and random coil structures were calculated from the integrated areas of the fitted peaks.

### 2.4 Preparation of pre-gelatinized rice flour

To gelatinize the rice flour, a rice-to-water ratio of 1:12 was used, and the mixture was heated at 90 °C for 45 min [19]. The resulting rice slurry was then spread onto trays and dried in a hot-air oven at 70 °C until a moisture content of 6–7% was reached [8]. Finally, the dried product was ground, passed through a 70-mesh sieve to obtain pre-gelatinized rice flour, and stored at room temperature until use.

### 2.5 Preparation of instant rice porridge (IRP)

IRPs were prepared by thoroughly dry blending 36.5% pre-gelatinized rice flour, 15% PPI, 14.2% skim milk powder (SMP), 14% whey protein concentrate (WPC 80), 12.5% sugar, 7.5% cream powder, and 0.3% vanilla powder. PPI was selected as the main protein source in the formulation. However, increasing the PPI level may result in undesirable sensory attributes. Therefore, protein fortification was designed based on both nutritional and sensory considerations. In addition, the PPI level was selected to provide the desired effects on the rheological and textural properties of the porridge. WPC and SMP were therefore used to increase the overall protein content and improve the sensory properties of the formulation.

The instant porridge containing NPPI was considered as the control sample. In the other formulations, NPPI was replaced with modified pea protein isolates. IRP samples containing PPI–PG (4:1), PPI–PG (2:1), UPPI 100/10, UPPI 100/20, UPPI 300/10, and UPPI 300/20 were coded as IRP4:1, IRP2:1, IRP100/10, IRP100/20, IRP300/10, and IRP300/20, respectively. For reconstitution, the powder mixtures were gradually added to distilled water (25 °C) at a water-to-powder ratio of 1.5:1 (w/w) and stirred continuously for 2 min to obtain a homogeneous dispersion. The reconstituted porridges were then left to stand for 5 min to allow hydration before analysis.

### 2.6 Characteristics of IRP

#### 2.6.1 Nutritional composition

The proximate composition of the control IRP powder was determined using standard AOAC methods [20], and carbohydrates were determined by difference. The energy value was calculated using Eq. (2) [21]:

*Energy value (Kcal/100g) = 4 (Protein) + 4 (Carbohydrate) + 9 (Fat)* (2)

#### 2.6.2 Water-holding capacity (WHC)

The WHC was determined according to the method of Xu et al. [22] with slight modification. Briefly, 15 g of reconstituted IRP was centrifuged at 8960 × g for 30 min at 25 °C, and the supernatant was carefully removed. The WHC was then calculated using Eq. (3):

*WHC (%) = (Weight of sample after centrifugation (g)/ initial weight of sample before centrifugation (g)) × 100 %* (3)

#### 2.6.3 Flow and viscoelastic properties

The rheological properties of reconstituted IRPs were assessed using an Anton Paar MCR301 rheometer (Anton Paar, Austria) featuring a 40 mm diameter parallel plate geometry and a gap of 1 mm at 25 °C. All measurements were done in triplicate [23].

##### 2.6.3.1 Flow rheological properties

Flow behavior was evaluated within a shear rate range of 0.01–100 s^−1^, and the apparent viscosity of the samples was measured at 25 °C [23]. The Herschel-Bulkley model (Equation (4)) was fitted to the flow curve data (Shear stress (τ) vs. Shear rate (γ̇) curves):

τ= τ *_0_* + *Kγ̇^n^* (4)

Where τ is the shear stress (Pa), τ_0_ is yield stress (Pa), *γ̇* is the shear rate (s^-1^), K is the consistency index (Pa.s^n^), and n is the flow index. In the Herschel–Bulkley model, the unit of K depends on n and is expressed as Pa.s^n^ when n ≠ 1.

##### 2.6.3.2 Viscoelastic properties

The viscoelastic properties of the reconstituted IRPs were characterized by oscillatory measurements, including strain sweep and frequency sweep tests, to determine the storage modulus (G’) and loss modulus (G”). The linear viscoelastic region (LVR) of each sample was first identified by a strain sweep test (0.01–100% strain at 1 Hz). A frequency sweep test was subsequently performed over the frequency range of 0.01–100 Hz at a constant strain of 0.1%, which was within the LVR [24].

#### 2.6.4 Textural properties

The textural properties of the reconstituted IRPs were evaluated using a texture analyzer (Stable Micro Systems, UK) by back extrusion and texture profile analysis (TPA) at 25 °C. All measurements for each sample were performed in triplicate.

##### 2.6.4.1 Back extrusion

A cylindrical container with a diameter of 60 mm was filled to 75 % of its height with 100 g of each sample. A plate probe, measuring 40 mm in diameter, was inserted into the sample with the following settings: 5 mm/s pre-test speed, 1 mm/s test speed, 5 mm/s post-test speed, distance 30 mm, and return distance 100 mm. Trigger force was set to 5 g [9]. Firmness, consistency, cohesiveness, and index of viscosity were derived from the back extrusion test.

##### 2.6.4.2 Texture profile analysis (TPA)

TPA was performed under the following test conditions: a 5 kg load cell, pre-test speed of 5 mm/s, test speed of 1 mm/s, post-test speed of 5 mm/s, a trigger force of 5 g, and compression of 35%. After the initial compression cycle, the probe was held for 5 s before the second compression [25]. Parameters, including hardness, cohesiveness, adhesiveness, and gumminess, were obtained from the TPA curve.

#### 2.6.5 Scanning electron microscopy (SEM)

The internal microstructure of the reconstituted IRPs was examined using a scanning electron microscope (Vega3, Tescan, Czech Republic). The reconstituted IRPs were spread onto glass slides and dried in an oven at 40 °C for 12 h. The cross-sectional morphology was observed at an accelerating voltage of 15 kV and magnifications of 200× and 500× [26], [27].

#### 2.6.6 IDDSI test methods

The IDDSI syringe flow, fork pressure, fork drip, and spoon tilt tests were performed to classify the reconstituted IRPs according to the IDDSI framework [7]. For the syringe flow test, 10 mL of sample was loaded into a syringe, and the remaining volume after 10 s was recorded. During the fork pressure test, a fork was pressed against the surface of the sample, and its deformation was evaluated. For the fork drip test, the sample was lifted with a fork, and both the amount of sample retained on the fork and its flow behavior at the fork tip were assessed. The spoon tilt test was performed to evaluate sample adhesion and cohesiveness. The sample was placed on a spoon, which was then tilted to observe its release behavior from the spoon surface.

#### 2.6.7 Sensory Evaluation

Sensory properties of the prepared samples were evaluated by 15 panelists (3 males and 12 females, aged 25–51 years) who had no swallowing difficulties. Before the evaluation, the panelists were given an explanation of dysphagia, and the characteristics of each sensory attribute were explained prior to assessment. The panelists evaluated the samples for taste, odor, mouthfeel, texture acceptability, ease of swallowing, and overall acceptability using a 5-point hedonic scale, ranging from 1 (“dislike very much”) to 5 (“like very much”). Prior to evaluation, each sample was prepared by dispersing 10 g of instant porridge in 15 g of water at 25 °C. The mixture was stirred for 2 min to obtain a homogeneous texture and then allowed to stand for 5 min before assessment. Samples were served at room temperature in plastic cups labeled with random three-digit codes. To minimize order and carryover effects, the serving sequence was randomized, and panelists were instructed to rinse their mouths with water between samples.

### 2.7 Statistical analysis

All measurements were performed at least three times, and the results are presented as mean ± standard deviation. The data were analyzed using SPSS Statistics version 27. One-way analysis of variance (ANOVA) was performed, followed by Duncan's multiple range test to determine significant differences (P < 0.05).

## 3 Results and discussion

### 3.1 Characteristics of the modified PPI

#### 3.1.1 Particle size distribution and zeta potential

The surface characteristics of a particle determine its functional, physical, and structural properties, which are directly affected by the behavior of the distribution of particle sizes [28]. The changes in particle size distribution of PPIs are shown in Fig. 1a. The findings indicated that modification significantly affected the particle size distribution of PPIs (P < 0.05). The PPI–PG complexes exhibited larger particle sizes than NPPI, whereas UPPIs showed smaller particle sizes. The highest particle size was observed for PPI–PG (2:1), whereas the lowest value was observed for UPPI 300/10. Following the addition of PG, the average particle size increased significantly from 2303.66 nm for NPPI to 3999 and 4808 nm for PPI–PG (4:1) and PPI–PG (2:1), respectively (P < 0.05). The increase in particle size following PG incorporation and its further increase with increasing PG content may be related to interactions between PPI and PG [12]. Similar results were reported by Yang et al. [12], who observed an increase in the proportion of larger pea protein particles following flaxseed gum addition. The reduction in particle size observed after ultrasound treatment may be attributed to cavitation, shear force, and turbulence generated during the ultrasound treatment. Moreover, the energy input associated with ultrasound would disrupt non-covalent interactions within proteins, including hydrogen bonds, electrostatic interactions, and hydrophobic interactions, therefore, contributing to the reduction in particle size and an increase in surface area. [18]. At 300 W, prolonging the treatment time to 20 min led to a partial increase in particle size. Similar observations were reported by Rafiei et al. [29] and Narale et al. [30], who reported that ultrasound treatment initially decreased particle size, whereas re-aggregation may occur under prolonged or high-intensity sonication. Previous studies have suggested that exposure of buried hydrophobic groups during sonication may enhance intermolecular hydrophobic interactions and promote the formation of larger aggregates [29], [30]. Zhang et al. [31] also observed an increase in particle size under excessive US treatment and suggested that intense cavitation, particle collisions, local overheating, and the generation of highly reactive free radicals could promote the re-aggregation of previously dispersed protein aggregates. They further suggested that such over-processing may also favor protein oxidation and stronger hydrophobic interactions among buried regions exposed during cavitation, which may further contribute to protein association.

**Fig. 1:**
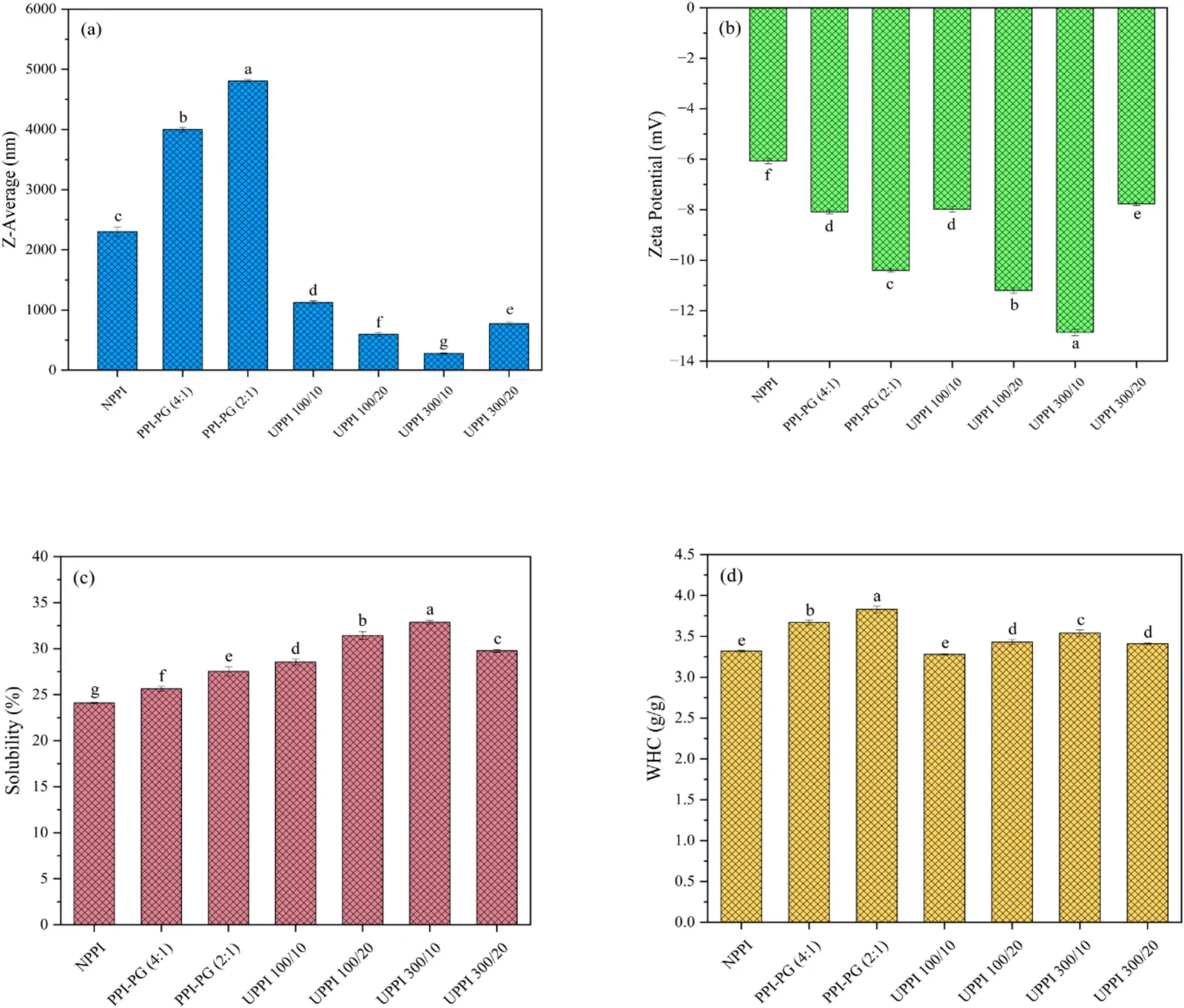
Average particle size (a), ζ-potential (b), solubility (c), and WHC (d) of native and modified PPI.

In addition, it has been reported that the cavitation and mechanical forces generated during ultrasound treatment may induce water-splitting reactions and generate highly reactive free radicals. These radicals may react with sulfur-containing amino acids and promote intermolecular disulfide cross-linking [32]. These processes have been suggested to contribute to protein aggregation under severe ultrasound conditions [31].

As illustrated in Fig. 1b, all samples exhibited negative zeta potential values. Among all samples, NPPI exhibited the least negative ζ-potential value, whereas UPPI 300/10 showed the most negative ζ-potential value. Compared with NPPI, the PPI–PG complexes exhibited more negative surface charges, with PPI–PG (4:1) and PPI–PG (2:1) showing values of −8.09 and −10.4 mV, respectively. The more negative ζ-potential observed with increasing PG content can be attributed to the greater contribution of negatively charged polysaccharide groups at the particle surface. The ζ-potential of PG is negative within the pH range of 3 to 7 [33]. Pea protein exhibits an anionic character at pH values above the pI due to the deprotonation of carboxyl groups in the presence of hydroxyl (OH^−^) ions [34]. Therefore, at pH 7, both PPI and PG possess negative charges. Mousazadeh et al. [33] suggested that, at pH 7, chickpea protein isolate (CPI) and the soluble fraction of Persian gum (SFPG) could interact despite their negative net charges. They suggested that localized positively charged patches on CPI may interact with the negatively charged carboxyl groups of SFPG. The authors also noted that hydrophobic interactions between non-polar regions of CPI and hydrophobic residues of SFPG may contribute to their association. A similar trend in ζ-potential was reported by Choi et al. [35] for pea protein–xanthan gum systems.

As shown in Fig. 1b, all UPPIs exhibited negative zeta potential values, suggesting the predominance of negatively charged amino acids on the protein surface [29]. At 100 W, increasing US treatment time led to an increase in ζ-potential. This effect is likely attributed to the greater exposure of negatively charged protein groups following sonication [36]. However, at 300 W, extending the treatment duration to 20 min reduced the magnitude of the ζ-potential. This may be associated with re-aggregation of protein particles during prolonged sonication, which could reduce the effective surface charge [36]. Similar results have been reported in previous studies by Narale et al. [30] and Kang et al. [36].

#### 3.1.2 Solubility

Solubility is an important indicator of protein hydration and strongly influences the functional properties and potential applications of proteins [36]. As shown in Fig. 1c, the PPI–PG and US-treated PPI samples exhibited significantly higher solubility than NPPI (P < 0.05). Solubility increased from 24.11 % in NPPI to 32.87 % in UPPI 300/10. Among the protein–polysaccharide complexes, PPI–PG (4:1) and PPI–PG (2:1) exhibited solubility values of 25.65 % and 27.53 %, respectively. The improved solubility may be attributed to the presence of hydrophilic polysaccharide chains, which enhance protein hydration and increase its affinity for water molecules [35]. Similar results have been reported by Choi et al. [35]. US treatment also enhanced PPI solubility. This enhancement can be attributed to ultrasound-induced conformational and structural modifications in the protein [28]. The cavitation and mechanical effects generated during sonication can depolymerize large protein aggregates into smaller particles [10]. As previously discussed, the reduction in particle size can increase the specific surface area. This may also expose more hydrophilic groups, thereby enhancing protein–water interactions [18]. Jiang et al. [37] also suggested that the dissociation of native protein complexes into individual subunits may be a driving force for increased protein solubility.

However, prolonged sonication at 300 W for 20 min resulted in a slight decrease in solubility. This may be attributed to the exposure of hydrophobic groups, which may promote protein–protein interactions and subsequent re-aggregation, thereby reducing solubility [38]. Similar findings were reported by Zhang et al. [38] and Kang et al. [36].

Overall, US treatment resulted in a greater increase in PPI solubility than PG complexation. This finding is consistent with the smaller particle sizes observed after US treatment, whereas the larger particle sizes of the PPI–PG complexes may have limited the extent of solubility enhancement.

#### 3.1.3 Water-holding capacity (WHC)

WHC reflects the ability of proteins to retain water through protein–water interactions and plays an important role in determining the texture, rheological properties, and overall quality of food products [39]. The WHC values of the native and modified PPI samples are shown in Fig. 1d. WHC was significantly affected by the modification treatments applied to PPI (P < 0.05). The highest WHC value, 3.83 g/g, was observed for PPI–PG (2:1). The enhancement in WHC following PG complexation may be attributed to the association of hydrophilic PG chains with protein molecules, thereby increasing their affinity for water molecules. Furthermore, PPI–PG (2:1) exhibited a higher WHC than PPI–PG (4:1), suggesting that the higher PG content could promote protein–water interactions and enhance water retention [40]. UPPI 100/10 showed no significant difference compared with NPPI (P > 0.05), similar to the results reported by Bahmanyar et al. [18] for ultrasonicated pea protein isolate. Meanwhile, the WHC of the remaining ultrasound-treated PPIs was significantly higher than of NPPI (P < 0.05). This improvement may be attributed to enhanced protein–water interactions associated with the reduced particle size and increased specific surface area following ultrasound treatment. These changes may promote water retention and increase WHC [41]. Similar observations were reported by Narale et al. [30] and Rafiei et al. [29].

Moreover, comparison of PG complexation and US treatment revealed that the effect of PG complexation on WHC was greater than US treatment. This difference may be attributed to the intrinsic water-absorption capacity of PG. PG is a highly branched polysaccharide and has been reported to absorb approximately 12 g of water per gram of gum, indicating its high water-absorption capacity [42], [43]. Therefore, incorporation of PG may provide additional water-binding sites within the modified PPI. In contrast, US treatment may expose hydrophilic groups and modify the PPI structure, thereby increasing its interaction with water [18]. Thus, the higher WHC of PPI–PG complexes can be attributed to the additional water-binding capacity of PG.

#### 3.1.4 Fourier-transform infrared spectroscopy (FTIR)

The secondary structural features of the PPIs were evaluated using FTIR spectroscopy (Fig. 2a). The FTIR spectra exhibited a broad band at 3700–3200 cm^−1^ attributed to O–H/N–H stretching vibrations, also amide I and amide II bands located in the regions of 1700–1600 and 1575–1480 cm^−1^, respectively [18], [39]. Following PG incorporation, shifts in the characteristic bands of the NPPI were observed. NPPI showed an O–H/N–H stretching band at 3288 cm^−1^, which shifted to 3269 cm^−1^ in the PPI–PG (4:1) sample, suggesting enhanced hydrogen-bonding interactions [44]. The PPI–PG (2:1) sample exhibited changes in the O–H/N–H stretching region, consistent with alterations in hydrogen-bonding interactions [18]. Overall, these spectral changes suggest that the association between PPI and PG may involve non-covalent interactions, particularly hydrogen bonding [44]. Similar observations have been reported by Yang et al. [12]. The amide I region provides valuable information on the conformational organization of proteins and is widely used to characterize changes in secondary structure following protein modification. In this region, α-helix, β-sheet, β-turn, and random coil structures are mainly assigned to 1646–1664, 1610–1637 and 1682–1700, 1664–1681, and 1637–1645 cm^−1^, respectively [45]. As shown in Fig. 2b, NPPI exhibited 34.14% α-helix, 36.26% β-sheet, 11.76% β-turn, and 17.83% random coil structures. Incorporation of PG markedly decreased the α-helix content to 21.07% and 22.67% in the PPI–PG (4:1) and PPI–PG (2:1) complexes, respectively. The pronounced reduction in α-helical structures suggested disruption of the native helical conformation and partial unfolding of the protein chains following PG incorporation [46]. Nevertheless, the subsequent structural rearrangement varied with the PPI-to-PG ratio. In the PPI–PG (4:1) complex, the loss of α-helix was accompanied by a marked increase in β-turn structures, from 11.76% in NPPI to 24.30%. In comparison, the PPI–PG (2:1) complex showed a pronounced increase in β-sheet structures, rising from 36.26% to 49.37%. These findings suggested that partial unfolding of PPI was followed by conformational rearrangement, with the resulting secondary-structure distribution depending on the relative proportions of PPI and PG.

**Fig. 2:**
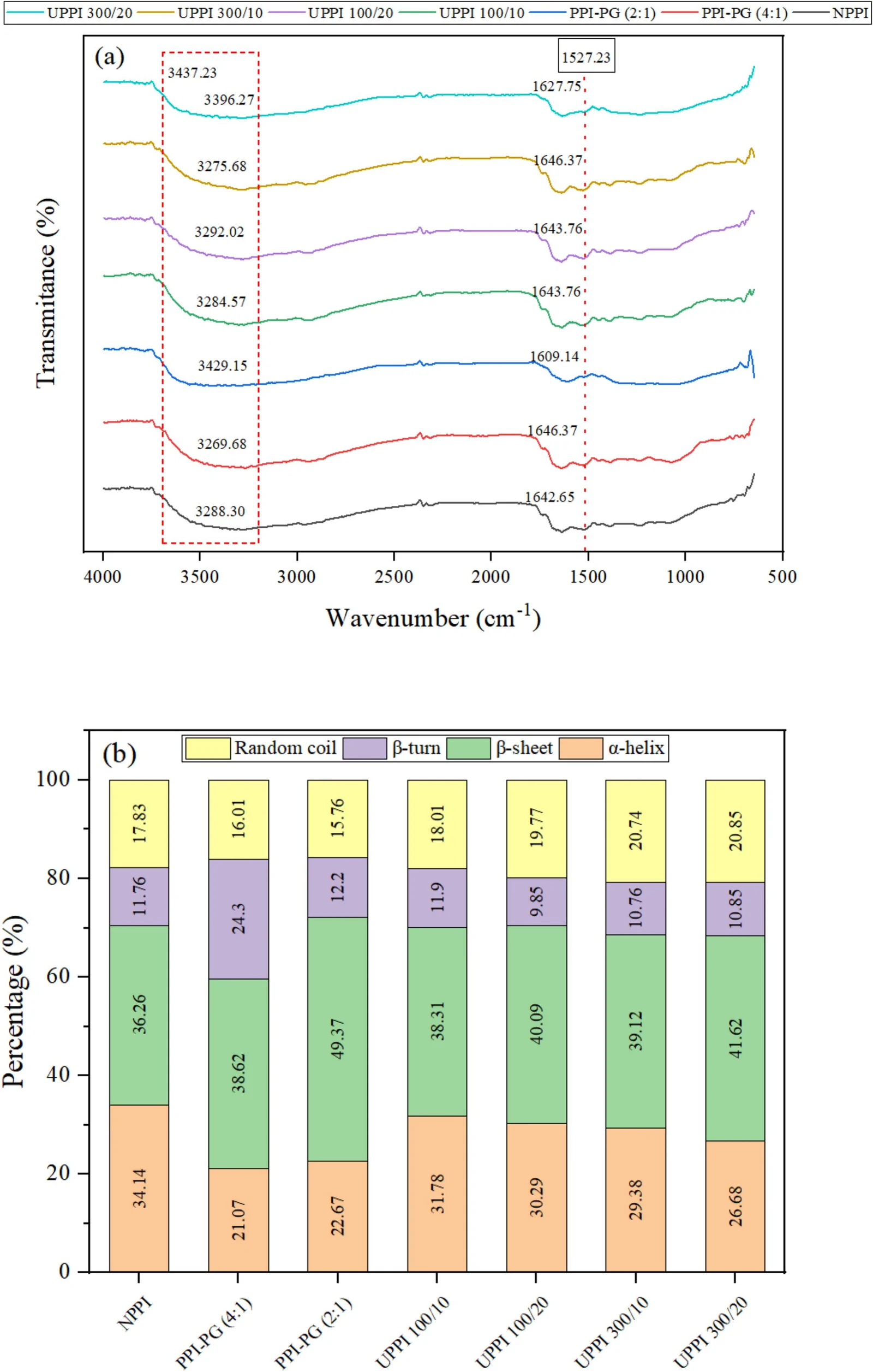
FTIR spectra (a) and secondary structures (b) of native and modified PPI.

For the UPPIs, the FTIR spectra indicated that structural modifications were dependent on ultrasound conditions. At 100 W, only minor shifts were observed in the O–H/N–H stretching region, while the amide I and II bands remained largely unchanged, indicating limited structural modifications. At higher ultrasonic power (300 W, 10 min), the amide I band shifted to 1646 cm^−1^, suggesting changes in protein conformation associated with partial unfolding and alterations in intramolecular interactions [30]. As shown in Fig. 2b, ultrasound treatment progressively altered the secondary structural organization of PPI, as reflected by a decrease in α-helix content and an increase in β-sheet content with increasing power and treatment time. These changes became more pronounced at 300 W. The reduction in α-helix content suggests destabilization of the native ordered conformation of PPI, which may result from the disruption of intramolecular hydrogen bonds involved in maintaining the protein structure [31]. However, at 300 W for 20 min, the amide I band shifted to 1627 cm^−1^, while two distinct peaks appeared in the O–H/N–H stretching region at 3396 and 3437 cm^−1^. These changes suggested more pronounced conformational changes under prolonged ultrasound treatment. Consistent with these changes, the β-sheet content of UPPI 300/20 increased from 36.26% to 41.62%. This increase may reflect the rearrangement of partially unfolded protein chains toward more ordered structures, which could be associated with the exposure of hydrophobic and sulfhydryl groups [30]. These observations are consistent with the increased particle size of UPPI 300/20 and with previous findings reported by Narale et al. [30].

### 3.2 Analyses on Instant Rice Porridge

#### 3.2.1 Nutritional composition

The energy value and nutrient composition of the control IRP sample are presented in Table 1. The recommended daily protein intake for older adults is approximately 1.2 g/kg body weight/day [9]. Therefore, protein enrichment was considered an important objective during formulation development. The protein content of the instant control powder was 31.29 g per 100 g. Since protein contributed more than 20% of the total energy value, the formulation meets the criteria for the “high protein” nutrition claim in accordance with Regulation (EC) No. 1924/2006 [47]. Swallowing difficulties may reduce food intake and contribute to inadequate energy and protein intake. Therefore, foods with higher energy and protein density could be useful for older adults with swallowing difficulties [48].

**Table 1:** Nutritional composition and energy value of 100 g control instant rice porridge powder.

Index	Control
Moisture (g)	5.01 ± 0.06
Protein (g)	31.29 ± 0.34
Fat (g)	3.83 ± 0.12
Carbohydrate (g)	56.64
Fiber (g)	1.8 ± 0.3
Ash (g)	1.43 ± 0.07
Energy (KJ/Kcal)	1621/386

Mean values ± standard deviation.

#### 3.2.2 Water-holding capacity (WHC)

The WHC of all reconstituted porridges is presented in Fig. 3. Both PPI–PG complexes and UPPIs significantly increased the WHC of the reconstituted porridges compared with the control sample (P < 0.05). The highest WHC values were observed for IRP300/10 and IRP300/20, increasing from 68.43 % in the control sample to 92.86 % and 93.84 %, respectively. IRP4:1 and IRP2:1 samples exhibited WHC values of 71.49 % and 78.80 %, respectively. The increase in the WHC of PPI–PG-containing porridges may be attributed to the hydrophilic nature of PG and its numerous hydroxyl groups, which facilitate water absorption and retention within the network structure [49]. Compared with PPI–PG complexes, the incorporation of UPPIs further increased the WHC of the porridges. Ultrasound cavitation can induce protein expansion and conformational changes. These changes may expose previously buried functional groups and facilitate interactions of UPPIs with water and other macromolecules, possibly through hydrogen bonding and electrostatic interactions within the porridge matrix. Such interactions may promote the formation of a denser and more stable three-dimensional network than that formed by the PPI–PG complexes, thereby enhancing water entrapment and reducing water loss during centrifugation [22], [50]. Similar effects have also been reported by Xu et al. [22].

**Fig. 3:**
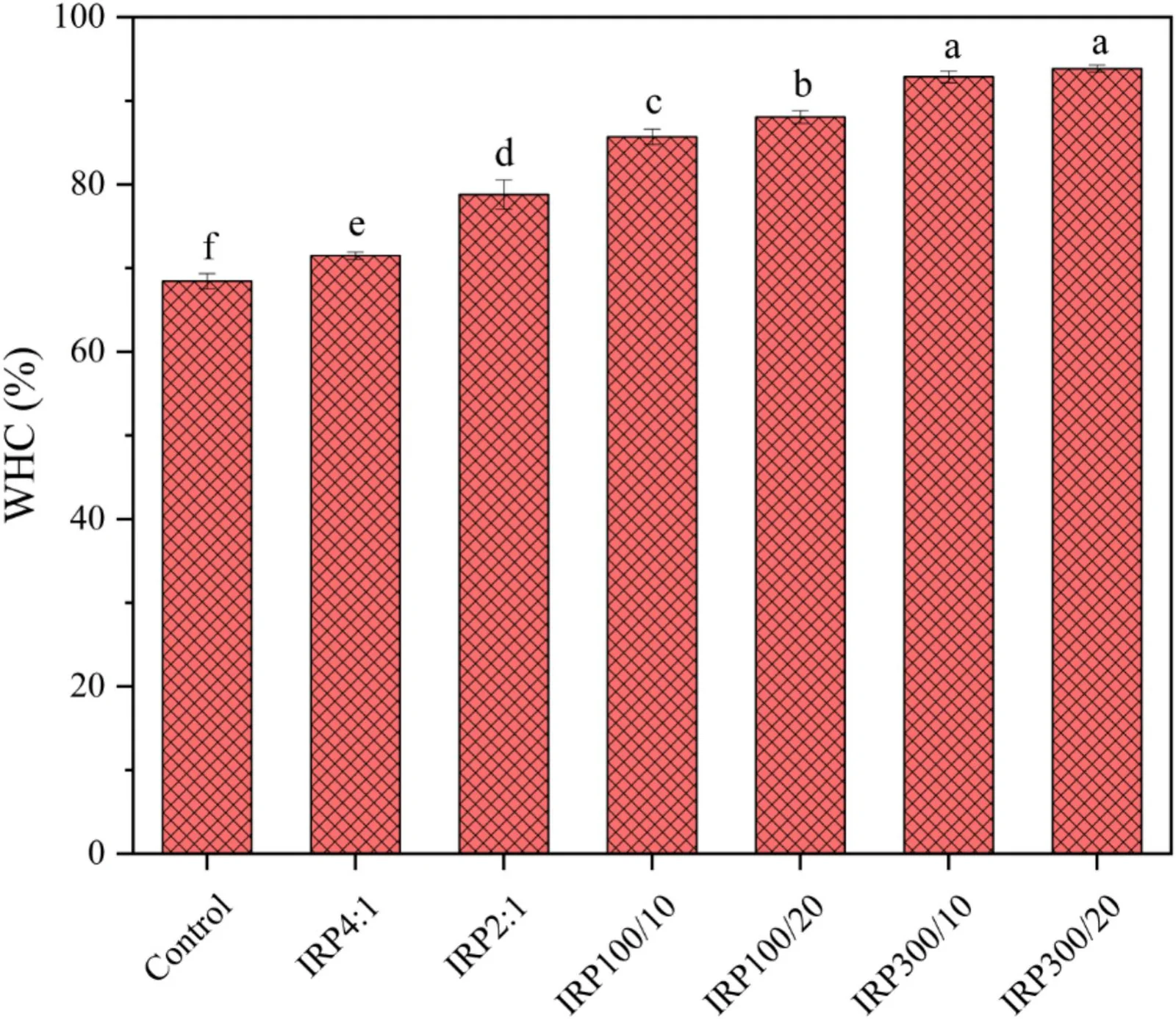
Water holding capacity (WHC) of reconstituted instant rice porridges.

Notably, among the modified PPI samples, the PPI–PG complexes showed higher WHC than UPPIs, as discussed in Section 3.1.3. However, UPPIs were more effective in increasing the WHC of the reconstituted porridges. This difference suggests that the WHC of the reconstituted porridges depends not only on the WHC of the modified PPI samples, but also on their contribution to the overall structure of the porridge matrix through interactions with other formulation components. In this regard, UPPIs appear to contribute more effectively to the development of a more structured porridge matrix, potentially enhancing water entrapment and, consequently, WHC. This interpretation is consistent with the higher firmness and hardness observed for UPPI-containing porridges. This improved water retention is particularly beneficial for patients with dysphagia, as it facilitates bolus lubrication during esophageal transit [22].

#### 3.2.3 Flow and viscoelastic properties

##### 3.2.3.1 Flow rheological properties

Fig. 4a illustrates the flow properties of the reconstituted rice porridges. All samples showed non-Newtonian flow with pseudoplastic (shear-thinning) behavior. Such rheological properties could be beneficial for individuals with dysphagia because this type of food can slow the swallowing process and facilitate the swallowing of a small amount of bolus [17]. All porridges containing MPPI exhibited significantly higher apparent viscosity than the control at all shear rates (P < 0.05). Among the PPI–PG formulations, IRP2:1 exhibited higher apparent viscosity than IRP4:1, which may be attributed to its higher PG content, leading to greater interactions between PG and water and contributing to the higher apparent viscosity [49]. The apparent viscosity of porridges prepared with UPPIs varied according to the ultrasound processing conditions. Elderly individuals with weakened pharyngeal muscles are unable to generate high shear rates comparable to those of healthy adults [17]. Therefore, the flow behavior of the formulations was evaluated at both low and high shear rates. At low shear rates (less than 10 s^−1^), the UPPI-containing porridges exhibited higher apparent viscosity than the PPI–PG formulations (IRP4:1 and IRP2:1) (Fig. 4a). The higher apparent viscosity of the UPPI-containing porridges at low shear rates may be attributed to the structural modifications induced by ultrasound, which likely promoted the formation of a more interconnected 3D network within the porridge matrix. Such structural changes may have restricted molecular mobility and increased resistance to flow, thereby contributing to the higher apparent viscosity [22]. Interestingly, the porridges prepared with UPPIs exhibited a more pronounced decrease in apparent viscosity with increasing shear rate compared with porridges containing PPI–PG complexes. This behavior was reflected by the steeper slopes of the flow curves for UPPI-containing porridges in Fig. 4b, indicating a stronger shear-thinning behavior. This suggested that, as the shear rate increased, the 3D network associated with UPPI-containing porridges became more susceptible to disruption by hydrodynamic forces, resulting in structural breakdown and a corresponding decrease in apparent viscosity [51]. In contrast, the higher apparent viscosity of porridges containing PPI–PG complexes at high shear rates may be related to the polymeric characteristics of PG. Its high molecular weight and branched structure may contribute to chain entanglement and hydration within the matrix [16], [52], thereby reducing the extent of viscosity decrease with increasing shear rate.

**Fig. 4:**
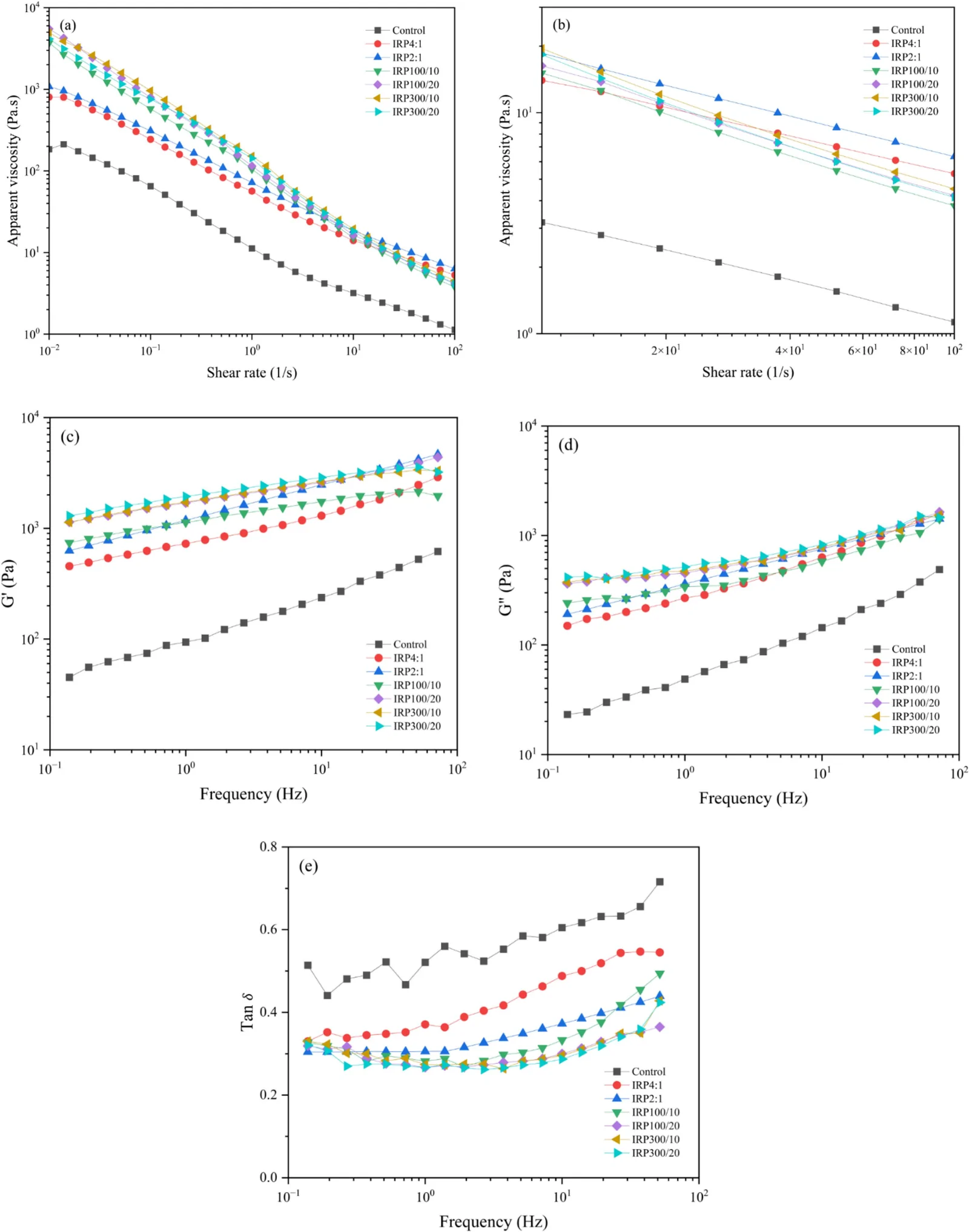
Apparent viscosity measured over shear rate ranges of 0.01–100 s^−1^ (a) and 10–100 s^−1^ (b), and frequency dependence of storage modulus (G’) (c), loss modulus (G”) (d), and loss tangent (Tan δ) (e) of reconstituted instant rice porridges at 25 °C.

Additionally, the apparent viscosity at 50 s^−1^, corresponding to the shear rate during swallowing, was also evaluated at 25 °C. At this shear rate, the control sample exhibited the lowest apparent viscosity (1.42 Pa·s), whereas IRP2:1 showed the highest value (8.42 Pa·s). All modified formulations exhibited apparent viscosity values greater than 1750 mPa·s. According to the National Dysphagia Diet (NDD) guidelines, the control sample was classified as honey-like (351–1750 mPa·s), whereas all modified formulations were classified as pudding-like (>1750 mPa·s) [53].

As presented in Table 2, the shear-thinning behavior of the formulations was fitted well by the Herschel–Bulkley model (0.973 < R^2^ < 0.999). The flow behavior index (n) values of all IRPs were lower than 1, indicating their pseudoplastic (shear-thinning) behavior. Among the modified formulations, IRP300/20 exhibited the lowest n value (0.337), whereas IRP4:1 showed the highest value (0.588). Compared with the PPI–PG formulations, the UPPI-containing porridges exhibited significantly lower n values, indicating more pronounced shear-thinning behavior, as observed in the flow curves. Moreover, food products exhibiting stronger shear-thinning behavior have been associated with a less sticky mouthfeel during oral processing. This characteristic can improve the sensory experience of patients with dysphagia [51]. Accordingly, porridges prepared with UPPIs may provide more favorable oral processing characteristics than the control and PPI–PG formulations. In addition, the consistency coefficient (K) increased in all modified formulations compared with the control. The highest K value was observed for IRP300/20 (84.570 Pa·s^n^). Yield stress (τ_0_) is another important rheological parameter for dysphagia-oriented foods because it represents the minimum stress required to initiate bolus flow during swallowing [1]. The control sample exhibited the lowest τ_0_ value (3.320 Pa), whereas all modified formulations showed significantly higher τ_0_ values (P < 0.05). Furthermore, the UPPI-containing porridges exhibited higher τ_0_ values than the PPI–PG formulations, suggesting that a greater stress was required to initiate flow. This finding is consistent with the firmer structure of the UPPI-containing porridges. This was reflected in their higher hardness and firmness values obtained from texture analysis.

**Table 2:** Flow and viscoelastic properties of the reconstituted instant rice porridges.

	Flow rheological properties					Viscoelastic properties		
Sample	η_50_ (Pa.s)	τ_0_ (Pa)	K( Pa.s^n^)	n	R^2^	G’ (Pa)	G’’ (Pa)	Tan δ
Control	1.42 ± 0.07^f^	3.320 ± 0.381^d^	7.739 ± 0.331^d^	0.578 ± 0.009^ab^	0.998	93.90 ± 8.30^e^	48.90 ± 5.11^f^	0.520 ± 0.008^a^
IRP4:1	6.88 ± 0.23^b^	14.725 ± 1.345^c^	34.197 ± 1.157^c^	0.588 ± 0.007^a^	0.999	726.00 ± 12.20^d^	269.00 ± 19.00^e^	0.370 ± 0.020^b^
IRP2:1	8.42 ± 0.18^a^	15.733 ± 1.616^c^	49.531 ± 1.485^b^	0.546 ± 0.006^b^	0.999	1181.00 ± 33.00^c^	361.00 ± 11.00^c^	0.306 ± 0.000^c^
IRP100/10	5.34 ± 0.14^e^	39.273 ± 4.999^b^	49.883 ± 5.464^b^	0.405 ± 0.023^d^	0.987	1130.00 ± 47.00^c^	319.00 ± 16.00^d^	0.282 ± 0.002^d^
IRP100/20	5.90 ± 0.11^d^	68.896 ± 4.078^a^	35.156 ± 4.059^c^	0.493 ± 0.025^c^	0.988	1700.00 ± 110.00^b^	455.00 ± 20.00^b^	0.267 ± 0.005^d^
IRP300/10	6.37 ± 0.10^c^	69.887 ± 8.250^a^	54.540 ± 9.007^b^	0.407 ± 0.035^d^	0.973	1720.00 ± 94.00^b^	472.00 ± 17.00^b^	0.274 ± 0.005^d^
IRP300/20	5.87 ± 0.13^d^	48.563 ± 11.592^b^	84.570 ± 13.129^a^	0.337 ± 0.031^e^	0.974	1940.00 ± 90.00^a^	517.00 ± 18.00^a^	0.266 ± 0.003^d^

Mean values ± standard deviation. Different letters indicate significant differences among formulations based on Duncan's post hoc test (p < 0.05). The apparent viscosity at a shear rate of 50 s^−1^ (η_50_), yield stress (τ_0_), consistency index (K), and flow behavior index (n) were determined from the Herschel–Bulkley model. Storage modulus (G’), loss modulus (G”), and Tan δ were obtained from frequency sweep test at 1 Hz. Control: instant rice porridge with NPPI, IRP4:1: instant rice porridge with PPI-PG (4:1), IRP2:1: instant rice porridge with PPI-PG (2:1), IRP100/10: instant rice porridge with UPPI 100/10, IRP100/20: instant rice porridge with UPPI 100/20, IRP300/10: instant rice porridge with UPPI 300/10, and IRP300/20: instant rice porridge with UPPI 300/20.

##### 3.2.3.2 Viscoelastic properties

The rheological properties obtained from frequency sweep measurements of the IRP samples are presented in Fig. 4. All porridges exhibited storage modulus (G’) values higher than loss modulus (G”) over the entire frequency range. This behavior indicated predominantly elastic and gel-like properties, which are characteristic of foods intended for individuals with dysphagia [53]. Compared with the control, porridges prepared with MPPIs exhibited higher storage modulus (G’) values, indicating enhanced elasticity behavior. This enhancement in elasticity suggested the development of a stronger internal network capable of resisting mechanical stress, as reported by Xu et al. [22] for curdlan–pea protein gels. Table 2 summarizes the viscoelastic parameters determined at 1 Hz within the LVR. At 1 Hz, both G’ and G” were higher in porridges prepared with MPPIs than in the control, with IRP300/20 exhibiting the highest G’ (1940 Pa) and G” (517 Pa) values. In addition to G’ and G”, the loss tangent (Tan δ = G”/G’) provides further insight into the viscoelastic behavior of the samples and can be used as an indicator of energy loss during oral processing [53]. According to Ishihara et al. [54], Tan δ values below 1.0 are considered one of the rheological criteria for ensuring that foods intended for individuals with dysphagia. Such values are associated with sufficient smoothness and deformability during oral processing and safe swallowing. In the present study, all IRP samples exhibited Tan δ values below 1.0, indicating that all formulations met this rheological criterion for dysphagia-oriented foods. Among the modified formulations, porridges prepared with UPPIs showed significantly lower Tan δ values (0.266–0.282) than the PPI–PG formulations (0.306 and 0.370). No significant differences in Tan δ were observed among the UPPI-containing porridges, whereas a significantly lower Tan δ value was observed for IRP2:1 than for IRP4:1, consistent with its higher PG content.

#### 3.2.4 Textural properties

##### 3.2.4.1 Back extrusion

The acceptability of food for individuals with dysphagia is highly influenced by its texture [55]. The textural characteristics of the IRP samples determined by the back extrusion test are presented in Table 3. Compared with the control, porridges prepared with MPPIs exhibited significantly higher firmness, consistency, cohesiveness, and index of viscosity values (P < 0.05). Similar increases in back extrusion parameters were also reported by Giura et al. [56] for texture-modified dysphagia foods. Firmness, defined as the force required to compress a food sample to a predetermined percentage of its original height. It reflects the resistance of the sample to deformation and is commonly associated with food hardness [57]. The highest firmness values were observed for IRP300/10 (181.69 g) and IRP300/20 (193.66 g), suggesting greater resistance to deformation during oral processing. Among the UPPI-containing IRPs, samples prepared with UPPIs treated at 300 W exhibited significantly higher firmness than porridges prepared with UPPIs treated at 100 W. The higher firmness values observed for IRP300/10 and IRP300/20 suggested the development of a stronger network structure within the porridge matrix [57]. Consistency and index of viscosity exhibited similar trends among the modified formulations. In the UPPI-containing porridges, both consistency and index of viscosity increased with increasing US power and treatment time, and the highest values were observed for IRP300/20. Also, among the PPI–PG formulations, IRP2:1 showed significantly higher consistency and index of viscosity values than IRP4:1. The index of viscosity was calculated from the negative area under the force–time curve during probe withdrawal [9]. This parameter is defined as the work required to overcome the attractive forces between the probe and the sample [13]. It also provides an indication of sample cohesiveness and consistency [56]. Accordingly, the greater absolute values of the index of viscosity observed in the UPPI-containing porridges indicated greater resistance to probe withdrawal. This finding was consistent with their more organized structure and higher cohesiveness and consistency. Cohesiveness refers to the force holding the food matrix together and reflects the strength of its internal bonds [13]. The UPPI-containing porridges exhibited significantly higher cohesiveness than the PPI–PG formulations (P < 0.05). The greater cohesiveness suggests stronger internal bonding and a more stable bolus structure, which may contribute to reducing the risk of aspiration [56].

**Table 3:** Textural properties of the reconstituted instant rice porridges determined by back extrusion.

Sample	Firmness (g)	Consistency (g.sec)	Cohesiveness (g)	Index of Viscosity (g.sec)
Control	25.87 ± 2.22^e^	931.17 ± 52.15^g^	–33.13 ± 2.12^d^	−116.68 ± 9.03^g^
IRP4:1	47.21 ± 2.17^d^	1204.11 ± 17.50^f^	−73.39 ± 3.98^c^	−201.48 ± 16.88^f^
IRP2:1	50.36 ± 5.94^d^	1285.86 ± 11.42^e^	−80.46 ± 5.48^c^	−235.90 ± 17.90^e^
IRP100/10	117.40 ± 12.23^c^	3933.47 ± 33.07^d^	−148.79 ± 23.36^b^	−315.02 ± 14.51^d^
IRP100/20	131.70 ± 12.63^b^	4478.27 ± 45.08^c^	−167.32 ± 24.97^b^	−346.88 ± 13.71^c^
IRP300/10	181.69 ± 5.95^a^	5559.48 ± 58.17^b^	−195.60 ± 19.82^a^	−743.71 ± 14.48^b^
IRP300/20	193.66 ± 1.55^a^	5791.10 ± 36.13^a^	−201.92 ± 16.99^a^	−877.71 ± 25.54^a^

Mean values ± standard deviation. Different letters indicate significant differences among formulations based on Duncan's post hoc test (p < 0.05). Control: instant rice porridge with NPPI, IRP4:1: instant rice porridge with PPI-PG (4:1), IRP2:1: instant rice porridge with PPI-PG (2:1), IRP100/10: instant rice porridge with UPPI 100/10, IRP100/20: instant rice porridge with UPPI 100/20, IRP300/10: instant rice porridge with UPPI 300/10, and IRP300/20: instant rice porridge with UPPI 300/20.

##### 3.2.4.2 Texture profile analysis (TPA)

TPA was performed to further characterize the textural properties of the IRP samples (Table 4). Compared with the control, porridges prepared with MPPIs exhibited significantly higher hardness, cohesiveness, adhesiveness, and gumminess values (P < 0.05). Hardness followed a trend similar to the firmness values obtained from the back extrusion test. This is consistent with the enhanced structural integrity and network formation attributed to the presence of MPPIs. The cohesiveness values obtained by TPA further supported the improved structural organization of the modified formulations. This increase was consistent with the higher consistency and cohesiveness values obtained from the back extrusion analysis. It may be attributed to enhanced intermolecular interactions promoted by UPPIs and PPI–PG complexes, which could contribute to improved bolus stability during swallowing [56]. Adhesiveness describes the tendency of a food bolus to adhere to surfaces within the oral cavity and pharynx [58]. The greatest absolute values were observed for IRP300/10 (−248.26 g) and IRP300/20 (−266.81 g), which may limit the suitability of these formulations for individuals with dysphagia [51]. Gumminess, reflecting the effort required for bolus preparation in the oral cavity [58]. It also increased following protein modification. The ultrasound-treated formulations generally exhibited higher values than the PPI–PG formulations, particularly at higher ultrasound intensity. Similar increases in TPA parameters were reported by Xu et al. [59] in strawberry–pea protein gels prepared with ultrasound-modified pea protein.

**Table 4:** Textural properties of the reconstituted instant rice porridges determined by texture profile analysis (TPA).

Sample	Hardness (g)	Cohesiveness	Adhesiveness (g.sec)	Gumminess (g)
Control	20.20 ± 1.40^d^	0.64 ± 0.02^e^	−53.79 ± 7.90^e^	24.19 ± 0.73^e^
IRP4:1	42.72 ± 5.09^c^	0.72 ± 0.03^d^	−93.43 ± 12.58^d^	37.77 ± 5.64^d^
IRP2:1	51.52 ± 3.47^c^	0.73 ± 0.01^cd^	−110.10 ± 19.67^d^	43.41 ± 4.44^d^
IRP100/10	73.54 ± 5.61^b^	0.74 ± 0.01^cd^	−184.93 ± 16.14^c^	55.85 ± 11.06^c^
IRP100/20	82.90 ± 4.99^b^	0.76 ± 0.01^bc^	−235.64 ± 12.29^b^	67.22 ± 9.32^bc^
IRP300/10	93.62 ± 6.51^a^	0.79 ± 0.02^ab^	−248.26 ± 16.31^a^	73.48 ± 7.60^ab^
IRP300/20	102.23 ± 8.01^a^	0.82 ± 0.02^a^	−266.81 ± 17.96^a^	84.78 ± 5.16^a^

Mean values ± standard deviation. Different letters indicate significant differences among formulations based on Duncan's post hoc test (p < 0.05). Control: instant rice porridge with NPPI, IRP4:1: instant rice porridge with PPI-PG (4:1), IRP2:1: instant rice porridge with PPI-PG (2:1), IRP100/10: instant rice porridge with UPPI 100/10, IRP100/20: instant rice porridge with UPPI 100/20, IRP300/10: instant rice porridge with UPPI 300/10, and IRP300/20: instant rice porridge with UPPI 300/20.

Overall, the back extrusion and TPA results showed that incorporation of PPI–PG complexes and UPPIs increased the measured textural parameters. The effect was more pronounced for the UPPI-containing formulations. These findings were consistent with the WHC results, suggesting that ultrasound treatment altered the structure of PPI, which may have promoted the formation of a stronger internal structure within the reconstituted porridges.

#### 3.2.5 Scanning electron microscopy (SEM)

The cross-sectional microstructures of the oven-dried reconstituted IRP samples are shown in Fig. 5. The UPPI-containing porridges generally exhibited a more porous microstructure, whereas the control, IRP4:1, and IRP2:1 samples displayed denser and more compact structures. As discussed in Section 3.2.2, the UPPI-containing porridges exhibited higher WHC, indicating that water was more effectively immobilized within the network and therefore remained less mobile during oven drying [60]. In addition to the higher WHC, UPPIs promoted the development of a more organized network structure in the porridges. This was reflected in the corresponding increases in rheological and textural properties following US treatment. Consequently, the UPPI-containing formulations exhibiting stronger structural networks could have been better able to resist the stresses generated by water evaporation and capillary forces during oven drying. This may have allowed the network to remain mostly intact and maintain void spaces within the matrix [61]. In contrast, the control, IRP4:1, and IRP2:1 samples appeared to have weaker structural networks. These structures were less resistant to the internal stresses generated by water evaporation during oven drying. This could lead to structural packing and a denser, more compact microstructure [61].

**Fig. 5:**
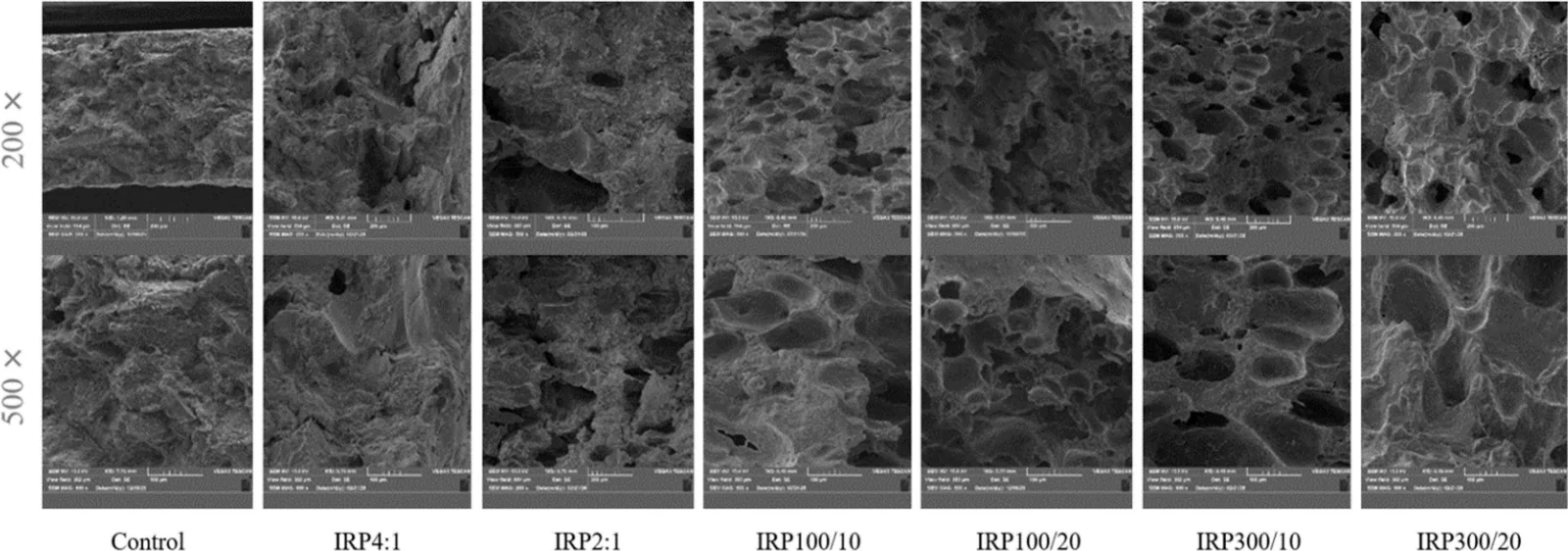
The cross-sections of the oven-dried reconstituted instant rice porridges at 200 × and 500 × magnifications.

#### 3.2.6 IDDSI Test Methods

The reconstituted rice porridges were evaluated according to the IDDSI framework to determine their suitability for dysphagia management. The results of the IDDSI assessment are presented in Fig. 6. During the syringe flow test, the control sample showed a remaining volume of approximately 9.6 mL, whereas the modified formulations did not flow through the syringe, leaving 10 mL after the test. These results indicate that the modified formulations exhibited higher resistance to flow compared with the control. In the subsequent fork pressure test, all samples deformed smoothly without whitening of the thumbnail and produced a surface pattern without obvious lumps. The fork drip test further revealed differences among the formulations. The control sample dripped through the gaps between the fork prongs, whereas IRP4:1 and IRP2:1 showed only a small amount of material passing through the prongs and forming a short tail below the fork. This behavior may be related to the weaker structural network of these formulations. In contrast, the UPPI-containing porridges did not flow through the gaps between the fork prongs. In the spoon tilt test, the control sample failed to maintain its shape on the spoon, whereas all modified formulations retained their shape. This was consistent with their higher cohesiveness compared with the control, as demonstrated by the previous textural analyses. Furthermore, IRP4:1 and IRP2:1 samples did not slide easily off the spoon and left noticeable residue on its surface. Similarly, IRP300/10 and IRP300/20 formulations also failed to slide readily, resulting in sample retention on the spoon. In contrast, the IRP100/10 and IRP100/20 samples left less residue on the spoon and slid off easily upon spoon tilting. This behavior is desirable for foods intended for individuals with dysphagia. Overall, the IDDSI findings were in agreement with the rheological and textural characteristics of the porridges.

**Fig. 6:**
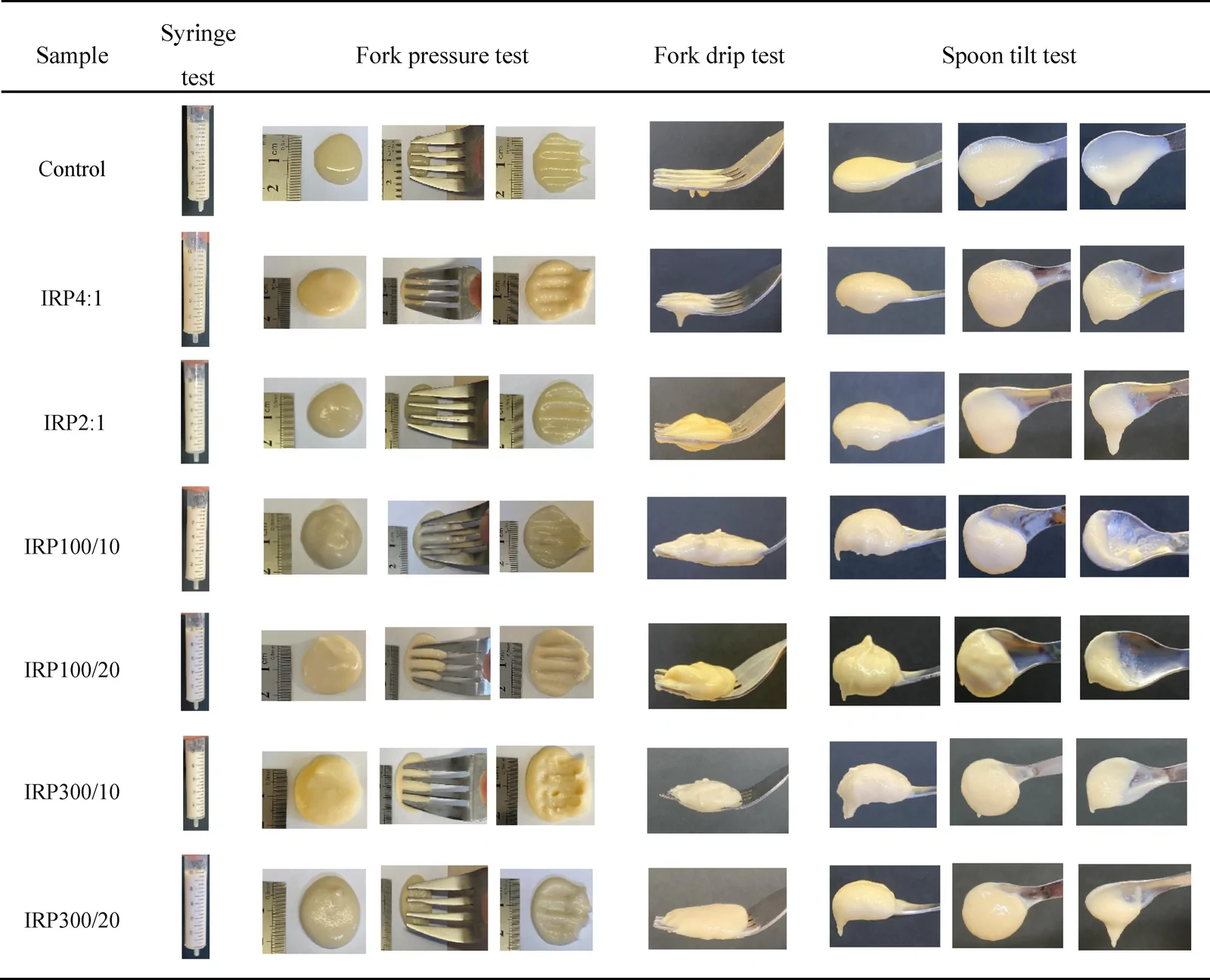
IDDSI test results of reconstituted instant rice porridges.

Based on the results of the IDDSI tests, the control sample was classified as IDDSI Level 3 (Liquidised/Moderately Thick). The PPI–PG formulations and the IRPs prepared with UPPI treated at 300 W failed the spoon tilt test and therefore did not meet the criteria for IDDSI classification. In contrast, IRP100/10 and IRP100/20 satisfied all IDDSI criteria and were classified as IDDSI Level 4 (Pureed).

#### 3.2.7 Sensory evaluation

The sensory attributes of the reconstituted rice porridges are shown in Table 5. No significant differences were observed between the control and the modified formulations in taste and odor (P > 0.05). No undesirable flavor was reported by the panelists, and the porridges were generally considered pleasant in taste and aroma. The addition of pre-gelatinized rice flour improved the rice aroma and sweetness of the IRPs, contributing to their overall palatability.

**Table 5:** Sensory attributes of reconstituted instant rice porridges.

Sample	Taste	Odor	Mouthfeel	Texture acceptability	Ease of swallowing	Overall acceptability
Control	3.6 ± 0.5^a^	4.0 ± 0.5^a^	1.3 ± 0.5^c^	1.6 ± 0.5^bc^	1.4 ± 0.5^b^	1.4 ± 0.5^b^
IRP4:1	3.8 ± 0.3^a^	4.0 ± 0.7^a^	1.6 ± 0.5^c^	1.7 ± 0.6^b^	1.4 ± 0.5^b^	1.6 ± 0.5^b^
IRP2:1	4.0 ± 0.0^a^	4.2 ± 0.4^a^	1.6 ± 0.5^c^	1.8 ± 0.6^b^	1.5 ± 0.5^b^	1.6 ± 0.5^b^
IRP100/10	4.1 ± 0.3^a^	4.3 ± 0.7^a^	3.6 ± 0.5^a^	3.7 ± 0.4^a^	3.7 ± 0.6^a^	3.7 ± 0.8^a^
IRP100/20	4.1 ± 0.6^a^	4.1 ± 0.6^a^	3.5 ± 0.5^a^	3.4 ± 0.5^a^	3.6 ± 0.5^a^	3.5 ± 0.7^a^
IRP300/10	3.8 ± 0.6^a^	4.1 ± 0.7^a^	2.3 ± 0.5^b^	1.2 ± 0.4^c^	1.3 ± 0.5^b^	1.3 ± 0.5^b^
IRP300/20	3.6 ± 0.5^a^	4.1 ± 0.6^a^	1.7 ± 0.4^c^	1.4 ± 0.5^bc^	1.2 ± 0.4^b^	1.1 ± 0.3^b^

Mean values ± standard deviation. Different letters indicate significant differences among formulations based on Duncan's post hoc test (p < 0.05). Control: instant rice porridge with NPPI, IRP4:1: instant rice porridge with PPI-PG (4:1), IRP2:1: instant rice porridge with PPI-PG (2:1), IRP100/10: instant rice porridge with UPPI 100/10, IRP100/20: instant rice porridge with UPPI 100/20, IRP300/10: instant rice porridge with UPPI 300/10, and IRP300/20: instant rice porridge with UPPI 300/20.

The IRP100/10 and IRP100/20 formulations received the highest scores for mouthfeel, texture acceptability, and ease of swallowing. These two formulations also achieved the highest overall acceptability among all samples. In contrast, porridges prepared with UPPI treated at 300 W received lower overall acceptability scores than those prepared with UPPI treated at 100 W. This was consistent with the back extrusion and TPA results. IRP300/10 and IRP300/20 showed greater firmness, hardness, and adhesiveness. Such characteristics may have contributed to a thicker oral consistency and reduced ease of swallowing, thereby negatively influencing sensory perception. Similarly, as discussed in the previous sections, the PPI–PG-containing porridges exhibited lower WHC and a less structured matrix. These characteristics may have negatively influenced mouthfeel and swallowing-related sensory attributes. In contrast, the swallowing scores of IRP100/10 and IRP100/20 were significantly higher than those of the other porridges (P < 0.05). This was in agreement with the IDDSI assessment. The findings indicate that MPPI incorporation significantly influenced the textural properties of the porridges and, consequently, their sensory perception (P < 0.05).

## 4 Conclusion

The present study aimed to modify PPI to improve its functional properties and evaluate its potential as a thickening agent in instant rice porridge. Both PPI–PG complexation and ultrasound treatment resulted in distinct changes in the structural and functional characteristics of PPI. Compared with NPPI, incorporation of PG increased particle size and surface charge. Also, PPI–PG complexes exhibited higher solubility and WHC than NPPI, likely associated with the hydrophilic nature of PG. Ultrasound treatment reduced particle size through cavitation and induced changes in the secondary structure of PPI, which increased its surface area and negative surface charge. These changes may have promoted protein–water interactions and improved its functional properties. However, at 300 W, extending the treatment time adversely affected these functional properties. This indicated that the effects of ultrasound treatment depended on both power and treatment time.

The MPPIs were subsequently applied as thickening agents in instant rice porridges. Nutritional characterization of the control sample revealed that its protein content was high enough for the product to be classified as “high protein”. The results further demonstrated that IRPs containing MPPIs exhibited significantly higher apparent viscosity, storage modulus, loss modulus, and superior textural properties than IRP containing NPPI (p < 0.05). In particular, the addition of UPPI promoted the formation of a stronger 3D network, which may have been associated with protein unfolding and interactions with other components. This was reflected in higher WHC and textural parameters, along with distinct rheological characteristics, compared with the PPI–PG-containing formulations. According to the IDDSI framework, IRP100/10 and IRP100/20 met all Level 4 (“Pureed”) criteria. They also received the highest sensory scores, supporting their palatability and acceptability.

Overall, the findings indicated that ultrasound-treated PPI, particularly under the 100 W treatment conditions, had potential as a thickening agent for improving the quality of instant rice porridges intended for dysphagia management. The results also suggested that the selection of ultrasound treatment conditions was important for obtaining desirable rheological, textural, and sensory properties.

## AI Declaration

During the preparation of this manuscript, the authors used ChatGPT (OpenAI) to improve the language, grammar, readability, and clarity of the manuscript. After using this tool, the authors reviewed and edited the content as needed and take full responsibility for the content of the published article.

## CRediT authorship contribution statement

**Yasamin Damroudi:** Writing – original draft, Methodology, Investigation, Formal analysis, Conceptualization. **Mehdi Farhoodi:** Writing – review & editing, Supervision, Project administration, Methodology, Conceptualization. **Leila Mirmoghtadaie:** Methodology, Investigation. **Kooshan Nayebzadeh:** Methodology, Investigation. **Fereshte Bahmanyar:** Writing – review & editing.

## Declaration of competing interest

The authors declare that they have no known competing financial interests or personal relationships that could have appeared to influence the work reported in this paper.
